# Robotic Heminephrectomy for Xanthogranulomatous Pyelonephritis in a Pediatric Patient

**DOI:** 10.7759/cureus.106590

**Published:** 2026-04-07

**Authors:** Mustapha Akhdar, Jami Zajicek, Carson Woodward, Abd El Rahman Abd El Barr

**Affiliations:** 1 Surgery, AdventHealth, Tampa, USA; 2 Pediatric Urology, AdventHealth, Tampa, USA

**Keywords:** nephrology, pediatric surgery, pyelonephritis, urology, xanthogranulomatous pyelonephritis

## Abstract

Xanthogranulomatous pyelonephritis (XGP) is a rare, chronic, destructive inflammatory condition of the kidney characterized by progressive renal parenchymal loss and replacement with lipid-laden macrophages, granulomatous inflammation, and fibrosis. In adults, XGP most commonly affects middle-aged women and is typically associated with urinary tract obstruction, nephrolithiasis, and recurrent infection. In contrast, pediatric XGP is exceptionally uncommon, with reported cases largely limited to isolated case reports and small case series. Pediatric presentations are frequently atypical, often lacking classic risk factors and instead manifesting with nonspecific symptoms or as an incidental renal mass. This creates a significant diagnostic dilemma, as focal XGP can radiographically mimic renal malignancy, abscess, or congenital renal pathology, with definitive diagnosis often established only after surgical resection.

We report the case of a 14-year-old male without a history of nephrolithiasis or recurrent urinary tract infections who was incidentally found to have a large left renal upper pole mass on computed tomography (CT) imaging obtained during evaluation for an unrelated condition. Imaging revealed a complex solid and cystic lesion measuring 11.5 × 7.9 × 10 cm with multiloculation, septations, perinephric inflammatory changes, and urothelial thickening, raising concern for malignancy versus complicated infection. Interventional radiology-guided nephrostomy tube placement drained purulent fluid, and cultures grew *Proteus* species, an organism commonly associated with XGP. Despite intravenous antibiotic therapy and drainage, repeat imaging demonstrated persistent lesion size and inflammatory burden. Given ongoing concern for a non-resolving renal mass, the patient underwent robotic-assisted left upper pole heminephrectomy. Intraoperatively, the upper pole was grossly abnormal with no viable renal parenchyma and a large abscess cavity. Final histopathologic evaluation confirmed focal XGP.

Although rare in children, XGP should remain on the differential diagnosis for complex renal masses, particularly when imaging demonstrates inflammatory or abscess-like features. This case highlights the diagnostic challenge of pediatric focal XGP and demonstrates the feasibility of a robotic-assisted nephron-sparing heminephrectomy approach in carefully selected patients. Early recognition of this entity may help guide timely surgical intervention while avoiding overtreatment for presumed malignancy and preserving functional renal tissue whenever possible.

## Introduction

Xanthogranulomatous pyelonephritis (XGP) is a rare, severe, chronic inflammatory disorder of the kidney characterized by progressive destruction of renal parenchyma and replacement by lipid-laden macrophages, multinucleated giant cells, and fibrosis. It is most commonly encountered in middle-aged women and is typically associated with long-standing urinary tract obstruction, nephrolithiasis, and recurrent bacterial infections, most frequently involving *Proteus* species or *Escherichia coli *[1].

In contrast, XGP is exceedingly uncommon in the pediatric population, with most reported cases limited to isolated case reports and small case series [1]. Pediatric presentations often differ from those seen in adults, with less frequent associations with renal calculi and more variable clinical manifestations, ranging from nonspecific constitutional symptoms to incidental radiographic findings. This variability, combined with the rarity of disease, makes preoperative diagnosis particularly challenging in children, as XGP can closely mimic renal neoplasms, abscesses, or congenital anomalies on imaging [2].

Radiographically, XGP may present as either diffuse or focal disease. Focal XGP, in particular, can appear as a mass-like lesion, raising concern for malignancy and often necessitating surgical exploration for definitive diagnosis. Histopathologic evaluation remains the gold standard for diagnosis [2]. Surgical management is typically required due to the destructive nature of the disease, with nephrectomy historically performed via an open approach. Advances in minimally invasive surgery have expanded the role of laparoscopic and robotic techniques in inflammatory renal conditions. However, data on minimally invasive management of pediatric XGP remain limited.

We present a rare case of focal XGP in a 14-year-old male presenting as an incidental renal mass, successfully managed with robotic upper-pole heminephrectomy. This case highlights the diagnostic challenges of pediatric XGP and demonstrates the feasibility of a robotic nephron-sparing approach in carefully selected patients.

## Case presentation

**Figure 1:**
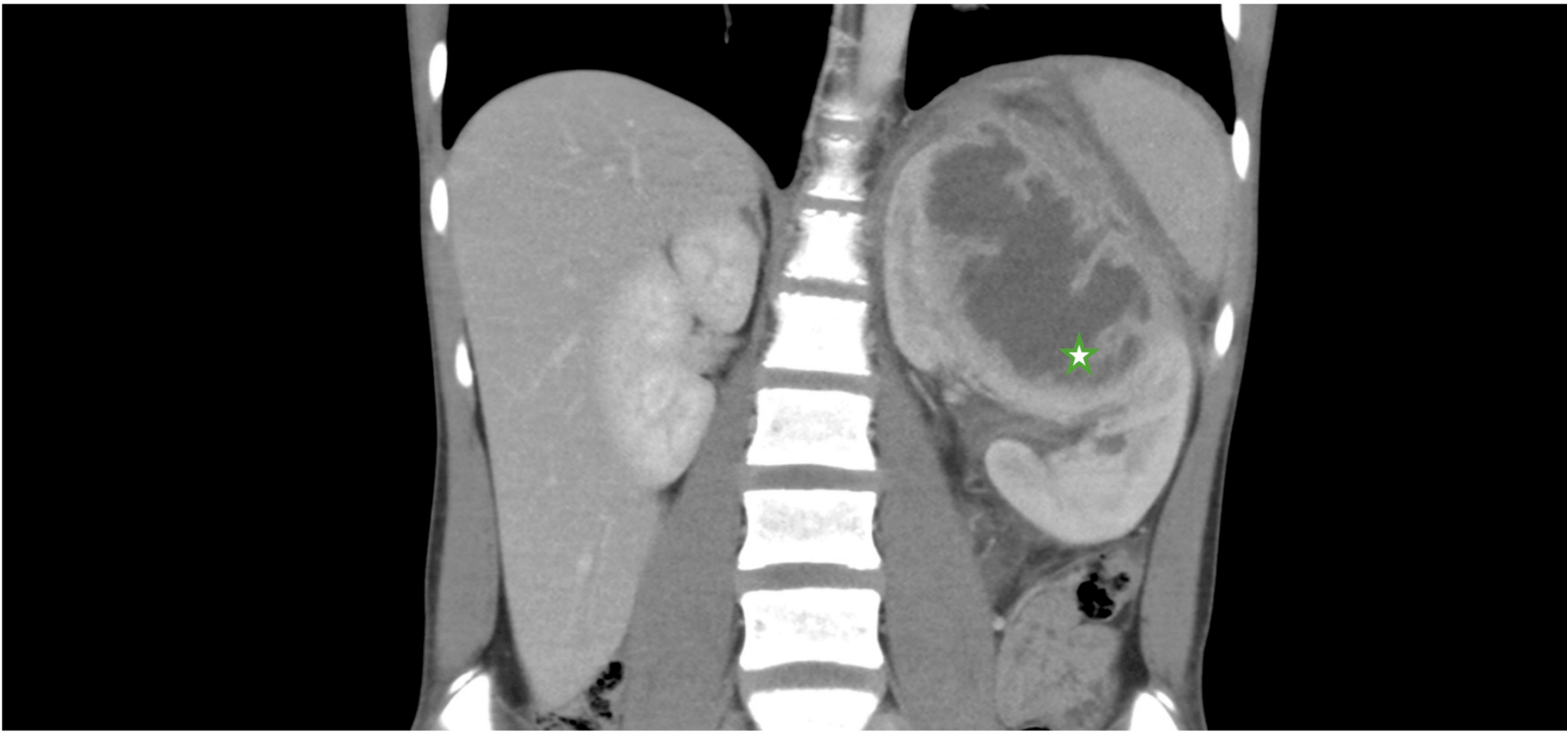
CT imaging (coronal view) Demonstrating a complex solid and cystic mass involving the upper to mid pole of the left kidney measuring 11.5 x 7.9 x 10 cm (star), with multiloculation, septations, perinephric micro-collections, extensive perinephric stranding and fluid, and enhancing urothelial thickening of the upper left renal collecting system.

**Figure 2:**
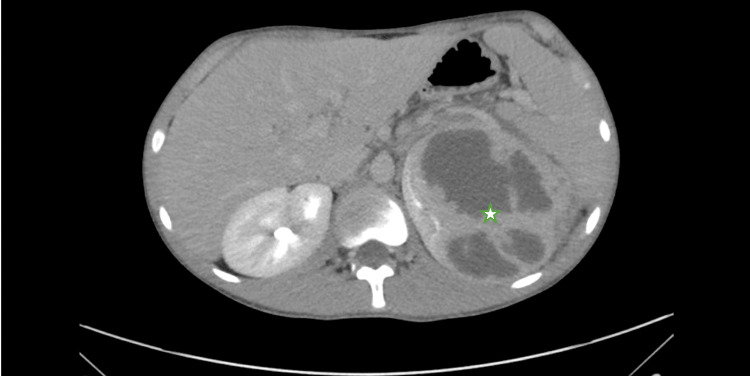
CT imaging (axial view) Demonstrating a complex solid and cystic mass involving the upper to mid pole of the left kidney measuring 11.5 x 7.9 x 10 cm (star), with multiloculation, septations, perinephric micro-collections, extensive perinephric stranding and fluid, and enhancing urothelial thickening of the upper left renal collecting system.

A 14-year-old male with a past medical history significant for pectus excavatum was incidentally found to have a partially visualized left renal mass on computed tomography (CT) imaging of the chest. Subsequent CT of the abdomen and pelvis was performed, which demonstrated an 11.5 x 7.9 x 10 cm complex solid and cystic mass involving the upper to mid pole of the left kidney (Figures 1, 2). The patient and his mother reported that two years ago, he experienced an episode of flank pain, fevers, and difficulty ambulating. He was evaluated in the emergency department at that time, where no significant pathology was identified. He received a single dose of antibiotics, after which his symptoms resolved. He denied any history of recurrent urinary tract infections, nephrolithiasis, weight loss, anorexia, malaise, or tuberculosis exposure. Given the incidental discovery of a renal mass in conjunction with multiple laboratory abnormalities (Table 1), the patient was admitted for comprehensive evaluation and further management.

**Table 1:** Pertinent laboratory findings on admission

Laboratory test	Value	Reference range
White blood cell count (WBC)	14.15 ×10^3/µL	4.0-11.0 ×10^3/uL
Hemoglobin	11.8 g/dL	12.0-16.0 (F) / 13.5-17.5 (M) g/dL
Platelet count	458 x 10^3/µL	150-400 x10^3/uL
Erythrocyte sedimentation rate (ESR)	>129 mm/hr	0-20 mm/hr
Sodium	136 mmol/L	135-145 mmol/L
Potassium	3.4 mmol/L	3.5-5.0 mmol/L
Blood urea nitrogen (BUN)	12 mg/dL	7-20 mg/dL
Creatinine	0.71 mg/dL	0.6-1.3 mg/dL
Aspartate aminotransferase (AST)	58 U/L	10-40 U/L
Alanine aminotransferase (ALT)	67 U/L	7-56 U/L
Alkaline phosphatase (ALP)	414 U/L	44-147 U/L
Total bilirubin	0.4 mg/dL	0.1-1.2 mg/dL
Total protein	9.5 g/dL	6.0-8.3 g/dL
Prothrombin time (PT)	15.5 seconds	11-13.5 seconds
International normalized ratio (INR)	1.19	0.8-1.1
Urinalysis	Unremarkable	_
Urine culture	No growth	_

On admission, interventional radiology performed placement of a percutaneous nephrostomy tube, which initially drained purulent fluid. Cultures obtained from nephrostomy drainage were positive for *Proteus* species. Anaerobic cultures and fungal cultures were negative. The patient was treated with ceftriaxone based on culture data and susceptibility.

Over the subsequent hospital course, nephrostomy output progressively decreased. Repeat imaging demonstrated a persistent left renal lesion without significant reduction in size despite drainage and antibiotic therapy. Given concern for an unresolved renal mass and inability to exclude malignancy, surgical intervention was pursued.

**Figure 3:**
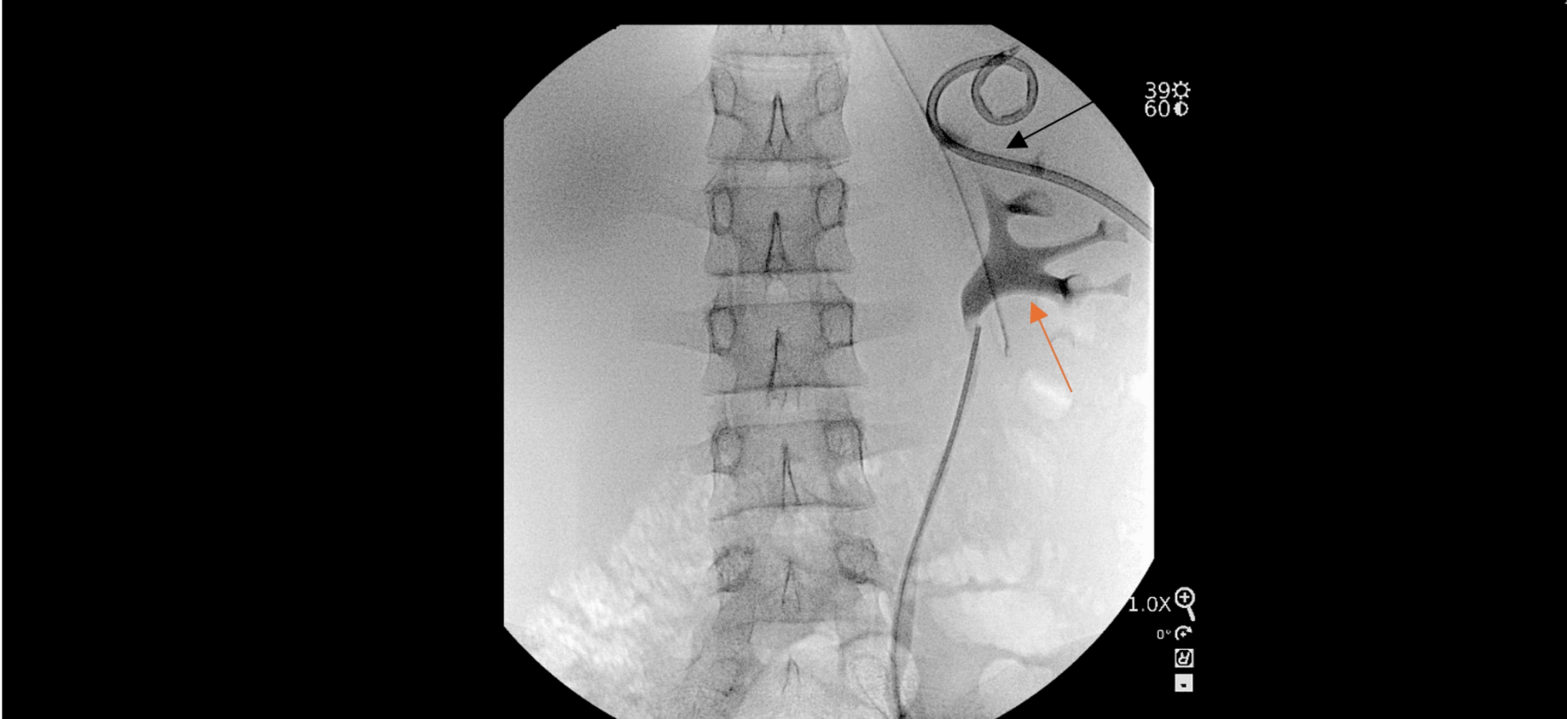
Intraoperative pyelogram Demonstrating an upper pole drainage catheter in place (black arrow). Inferiorly, a normal collecting system is visualized without evidence of duplication (red arrow).

The patient underwent robotic-assisted left upper pole heminephrectomy. Intraoperatively, retrograde pyelography demonstrated a normal collecting system without evidence of duplication (Figure 3). The lower pole renal parenchyma appeared viable and grossly normal, while the upper pole was markedly abnormal with no identifiable viable parenchyma. During dissection and separation of the upper pole tissue, a large abscess cavity was encountered, with intraoperative findings consistent with XGP. The postoperative course was uncomplicated, and the patient was discharged home on postoperative day five with a week of oral antibiotics. Final surgical pathology confirmed the diagnosis of XGP. At two-week postoperative follow-up, the patient was asymptomatic and recovering well.

## Discussion

XGP is predominantly described in adults, with pediatric cases representing only a small fraction of reported diagnoses. The rarity of XGP in children contributes to diagnostic delays and uncertainty, as clinicians may be less likely to include it early in the differential diagnosis. Additionally, pediatric patients often lack classic adult-associated risk factors such as staghorn calculi, chronic urinary obstruction, or recurrent documented urinary tract infections [1-3]. This case adds to the growing but still limited body of literature describing pediatric XGP and reinforces the importance of maintaining this entity in the evaluation of complex renal masses in children.

The pathogenesis of XGP is believed to involve chronic urinary obstruction and infection, resulting in impaired drainage, tissue ischemia, and a sustained inflammatory response. Progressive infiltration of lipid-laden macrophages and granulomatous inflammation ultimately replaces functional renal tissue. Isolation of *Proteus* species in our patient aligns with commonly reported pathogens implicated in XGP and supports the infectious component of disease progression.

Pediatric XGP frequently presents with vague or nonspecific symptoms, including abdominal or flank pain, fever, malaise, and weight loss, although cases may also be discovered incidentally, as in our patient. Focal XGP poses a particular diagnostic challenge, as it often presents as a mass-like lesion that closely mimics renal malignancy on imaging due to heterogeneous enhancement, cystic components, and associated inflammatory changes. Despite advances in imaging, reliable preoperative differentiation between focal XGP and neoplasm remains difficult, and diagnosis is frequently established only after surgical excision and histopathologic evaluation.

CT remains the imaging modality of choice in suspected XGP and typically demonstrates renal enlargement, heterogeneous parenchymal involvement, multiloculated cystic areas, perinephric stranding, and abscess formation. In focal disease, radiographic findings may be indistinguishable from renal tumors, and several reports in the literature emphasize that pediatric focal XGP is often diagnosed only after operative intervention. In our case, persistence of the lesion despite nephrostomy drainage and intravenous antibiotic therapy further supported surgical management in the setting of ongoing diagnostic uncertainty.

Laboratory evaluation in our patient was consistent with a significant inflammatory process, including leukocytosis (WBC 14.15 ×10³/µL), thrombocytosis (458 ×10³/µL), mild anemia (hemoglobin 11.8 g/dL), and markedly elevated inflammatory markers with an erythrocyte sedimentation rate (ESR) of >129 mm/hr. Importantly, renal function remained preserved (creatinine 0.71 mg/dL), supporting the feasibility of a nephron-sparing approach. Notably, despite extensive renal inflammation, urinalysis and urine culture were negative. This phenomenon has been described in prior reports, particularly in focal XGP, where infection and inflammatory destruction may remain localized to the affected renal segment and may not reliably communicate with the collecting system. As a result, urine studies may be falsely reassuring and should not exclude the diagnosis when clinical suspicion remains high. In our patient, nephrostomy tube drainage cultures were positive for *Proteus* species, further supporting an infectious etiology and reinforcing the diagnostic value of directed drainage cultures when available [4-6].

Definitive management of XGP is surgical, as medical therapy alone is typically insufficient due to extensive parenchymal destruction and persistent infection. While open nephrectomy has historically been favored because of dense inflammation and adhesions, minimally invasive approaches have increasingly been described with favorable outcomes in appropriately selected patients [7,8]. Robotic heminephrectomy has been well established in pediatric urology, particularly in the setting of duplex collecting systems. However, there remains limited published experience describing robotic nephron-sparing surgery for pediatric XGP. Our case demonstrates that robotic-assisted upper pole heminephrectomy can be safely performed in this setting, offering potential advantages including improved visualization, precise dissection in distorted tissue planes, reduced postoperative pain, and accelerated recovery, benefits that are particularly meaningful in the pediatric population [9,10]. Increased awareness of pediatric focal XGP is critical to guide timely surgical intervention, avoid misdiagnosis as malignancy, and preserve functional renal tissue whenever feasible.

## Conclusions

XGP is a rare and diagnostically challenging entity in the pediatric population and may closely mimic renal malignancy on imaging. Because clinical findings and urine studies may be nonspecific, definitive diagnosis often requires surgical resection with histopathologic confirmation. This case highlights that robotic-assisted nephron-sparing heminephrectomy is a feasible and effective option for focal pediatric XGP, offering a minimally invasive approach that may preserve functional renal tissue while achieving definitive source control. 
